# Self-Assembling Peptide Nanofiber Scaffolds Enhance Dopaminergic Differentiation of Mouse Pluripotent Stem Cells in 3-Dimensional Culture

**DOI:** 10.1371/journal.pone.0084504

**Published:** 2013-12-20

**Authors:** Na Ni, Yaohua Hu, Huixia Ren, Chuanming Luo, Peng Li, Jian-Bo Wan, Huanxing Su

**Affiliations:** State Key Laboratory of Quality Research in Chinese Medicine, Institute of Chinese Medical Sciences, University of Macau, Macao, China; University of Udine, Italy

## Abstract

Dopaminergic differentiation of embryonic stem cells (ESCs) gains more and more attention worldwide owing to its potential use for neurorestorative therapy for the treatment of Parkinson’s disease. The conventional 2D cell culture on petri dishes with various animal derived substrata such as collagen gels, laminin, and Matrigel is widely used to induce dopaminergic differentiation and it may limit the efficiency in the generation of dopaminergic neurons from ESCs and prevent their application for human therapies. Here, we reported that a self-assembling peptide made from natural amino acids has a property to generate a true 3D environment for dopaminergic differentiation. Mouse ESCs (R1) and mouse iPSCs (TTF-1) embedded in RADA16-I peptide-derived nanofiber scaffolds led to a marked increase in dopaminergic differentiation compared to the laminin-coated 2D culture or Matrigel-encapsulated 3D culture. These differentiated neurons expressed specific dopaminergic markers and produced appropriate patterns of action potential firing. Consistent with the increase in the number of dopaminergic neurons differentiated from R1 or TTF-1 in the self-assembling peptide nanofiber scaffold (SAPNS), both the expression levels of genes that involve in dopaminergic differentiation and maturation and the dopamine release in SAPNS culture were significantly elevated. The results of the study suggest that SAPNS provides a promising 3D culture system for dopaminergic differentiation.

## Introduction

Cell therapy holds great promise for the treatment of neurodegenerative diseases such as Parkinson’s diseases (PD) where pharmacological interventions or other treatment strategies are currently lacking. Of all stem cell types, embryonic stem cells (ESCs), which are derived from the inner cell mass of blastocysts, are considered to possess the greatest potential for the widest range of cell replacement therapies. A prerequisite for clinical application of ESCs in the treatment of PD is an efficient and strict differentiation of ESCs into midbrain dopaminergic neurons. In this regard, various strategies for improving efficiency of dopaminergic differentiation from ESCs have been developed for the past decade, mostly by optimizing culture conditions [1-6], manipulating genetic modification [7,8], and modulating intracellular signaling pathways [9-14].

Although these approaches have elegantly shown successful dopaminergic differentiation and led to higher yield of dopaminergic neurons, it should be noted that nearly all these studies used the conventional 2-dimensional (2D) tissue cell culture on various animal derived substrata such as collagen gels, laminin, poly-glycosaminoglycans and Matrigel to induce dopaminergic differentiation. The 2D tissue cell culture is different from the architecture of the in situ environment of cells in a living organism, which may affect the differentiation efficiency due to the changes in cellular growth and communication, nutrient transport and waste removal. Furthermore, the substrata used in these studies are animal derive and often contain residual growth factors, undefined constituents or non-quantified substances [15-17]. This makes it difficult to conduct well-controlled studies with these materials and prevents clinical application for human therapies. 

A self-assembling peptide system, which is made from natural amino acids and forms nanofiber scaffold hydrogels by altering salt concentration, represents a promising biomaterial for neural repair and 3D cell culture. It has excellent biocompatibility and biodegradability due to its naturally constituent amino acids and no cytotoxic and immunological alert after implantation. Our previous studies showed that self-assembling peptide nanofiber scaffolds (SAPNS) effectively facilitate brain and spinal cord repair in brain and spinal cord injury models and promote regeneration of peripheral nerves in a sciatic nerve injury model [18-20]. It can undergo spontaneous assembly into nanofiber scaffolds (10 nm in fiber diameter with pores between 5–200 nm) and surrounds cells in a manner similar to the natural extracellular matrix, thus producing a true 3D culture environment for cell growth, migration and differentiation [21-23]. The survival and differentiation of various kinds of cells such as neural stem cells, Schwann cells, and osteoblasts were greatly improved when cultured in SAPNS-derived 3D culture system [18,24-26]. 

However, it remains unknown whether ESCs can successfully differentiate into dopaminergic neurons in SAPNS and whether the efficiency of dopaminergic differentiation of ESCs can be improved in a 3D culture system. Therefore, the present study was designed to investigate the dopaminergic differentiation of mouse pluripotent stem cells including mouse ESCs and mouse induced pluripotent stem cells (iPSCs) in SAPNS-derived 3D culture system.

## Materials and Methods

### Cell culture

Mouse ESCs (R1) were from American Type Culture Collection (ATCC); Mouse iPSCs (TTF-1) were from our previous reported study [27]. All the cells were maintained on mitomycin-treated MEF feeder cell layers and cultured in KSR medium consisting of DMEM Dulbecco's Modified Eagle's Medium (DMEM; GIBCO, Invitrogen), supplemented with 20% knock-out serum replacement (KSR, GIBCO, Invitrogen), 2mM L-glutamine, 0.1mM nonessential amino acids, 0.1 mM β-mercaptoethanol, and 1000 U/ml leukemia inhibitory factor (LIF, Chemicon International) with the culture medium renewed every day.

### Dopaminergic induction

Dopaminergic neuron differentiation of mouse pluripotent stem cells followed a well-established protocol with minor modifications [1]. Firstly, R1 (passage 20-25) or TTF-1 cells (passage 15-20) were dissociated into single cells after removal of feeder cells. They were then grown at 0.5-1×10^5^ cells/ml in aggregate cultures in DFK10 medium to form EBs. DFK10 medium consisted of DMEM/F12 (Invitrogen) supplemented with 10% Knock-out serum replacement, 2.4% N2 (GIBCO), 4500 mg/l Glucose, 2mM L-glutamine, 1 u/μl Heparin (Sigma), and 0.1mM β-mercaptoethanol. EBs were formed in DFK10 medium for 4 days and then plated on laminin-coated plates. After 24h of culture, DFK10 medium was replaced by DFK10 supplemented with 1% Insulin/Transferrin/Selenium/Fibronectin (ITSFn, GIBCO) medium. After 6–10 days, neural rosettes were selected and dissociated by 0.05% trypsin/EDTA and finally adjusted to 5× 10^5^ cells/μL. The dissociated cells were subsequently processed to induce dopaminergic differentiation.

For SAPNS-derived 3D culture, RADA16-I peptide (BD Biosciences, Cambridge, MA) was used for encapsulating cells. Five microliters of freshly dissociated cells suspended in serum-free, bFGF-supplemented basal neural cell culture medium were mixed with 45 μL of the peptide in the wells of a 24-well plate. When the peptide solution was mixed with the cell suspension, gelation was initiated, resulting in cell encapsulation inside the nanofiber hydrogel. Around 500 μL of serum free basal medium supplemented with bFGF was added to neutralize the acidic hydrogel environment. The medium was changed at 1, 10, and 30 minutes after plating until the pH of the system was neutral. Then the medium was replaced with N2 medium supplemented with 10 ng/ml bFGF, 200 ng/ml SHH (R&D Systems) and 100ng/ml FGF8b (R&D Systems). After 7 days, the culture medium was changed to N2 medium supplemented with 1 μM cAMP (Sigma), 200 μM AA (Sigma), 10ng/ml BDNF (R&D Systems) and 10ng/ml GDNF (R&D Systems) and the cells were further cultured for 5–10 days.

Three-dimensional culture was also performed in Matrigel (BD Biosciences) which was served as a control group. Five microliters of freshly dissociated cells were mixed with 45 μL of Matrigel and then plated on the wells in a 24-well plate. Another control group was the dopaminergic differentiation in a conventional 2D culture condition in which 5 μL of cell suspension were directly plated on laminin-coated wells in a 24-well plate. 

### Immunocytochemistry study

Cells were fixed in 4% paraformaldehyde dissolved in 0.1 M phosphate buffer (PB) for 20 min. After several washes with 0.01 M phosphate-buffered saline (PBS), the cultures were incubated with the primary antibodies in PBS plus 1% BSA, 10% normal goat serum, and 0.3% Triton X-100 over night at 4°C. The following primary antibodies were used to stain the cells: monoclonal anti-Nestin (1: 500; Sigma) for R1 or TTF-1 cells-derived neural progenitors; polyclonal anti-neuronal class III β-tubulin (Tuj1) (Covance, Berkeley, CA) for early differentiating neurons; monoclonal anti-TH (1:500; Sigma), polyclonal anti-Nurr1 (1:500; Thermo Scientific), polyclonal anti-Dat (1:200; Bioss Inc) for dopaminergic neurons; polyclonal anti-dopamine-b-hydroxylase (Dbh) (1:400; Chemicon) for adrenergic neurons, and polyclonal anti-tryptophan hydroxylase 2 (Tph2) (1:500; Millipore) for serotonergic neurons. Primary antibodies were visualized with species-specific secondary antibody conjugated to the fluorescent labels Alexa 568 or 488 (1: 400; Molecular Probe, Eugene, OR, USA). Cells were mounted in anti-fade medium containing 4',6-diamidino-2-phenylindole (Sigma) to counterstain nuclei. To quantify dopaminergic differentiation, 8-9 representative fields per well were randomly selected and digitized images were obtained with a cooled CCD digital camera. Five wells per experiment were imaged. Cells were counted on images imported and processed using a semi-automatic stereology system with stereo investigator (MicroBrightField, Williston, VT). Results were mean ± SEM of data from five experiments unless stated otherwise in legends.

### Quantitative RT-PCR (qPCR)

Total RNAs of each group’s cells were extracted using TRIzol (Invitrogen) at different differentiation time points. Quantitative real time RT-PCR (qPCR) was performed using a Thermal Cycler DiceTM Real Time System and SYBR Premix EX TaqTM (Takara). The expression of a number of genes that involve in dopaminergic differentiation or maturation was investigated. Meanwhile, the expression of genes coding for adrenergic and serotonergic biosynthetic enzymes was also investigated. All primer sequences were listed in table S1. Standard curves and melting curves were determined for each set of primers to confirm that a single amplicon was generated. β-actin was used for qPCR normalization, and all items were measured in triplicate. Relative expression ratios were calculated by the ΔΔCt method.

#### Whole-cell patch-clamp recordings

Whole-cell patch-clamp recording techniques were used to study the intrinsic properties of dopaminergic neurons differentiated from R1 or TTF-1. Resting potentials were maintained at about -65 mV. Whole-cell patch- clamp techniques were amplified and filtered using an Axopatch 200B amplifier (Molecular Devices, Sunnyvale, CA). Graded current injections used durations of 300 milliseconds (in steps of 5 pA). Signals were sampled at 10 kHz using a Digidata 1440A analog to digital converter and acquired and stored on a computer hard drive using pClamp10 software. Data were analyzed using pClamp10 (Clampfit).

### Dopamine release measurement by HPLC

After 7 days of exposure to specific midbrain patterning factors (SHH and FGF8b) and followed by 10 days of maintenance with cAMP and neurotrophic factors, cells in SAPNS, Matrigel or on laminin were rinsed twice with Hanks’ balanced salt solution (HBSS) and then incubated in 200 μl of N2 medium supplemented with 56 mM KCl for 30 min. The media were then collected and stabilized with 0.1 mM EDTA and analyzed for dopamine level using an HPLC coupled to an ESA Coulochem II Detector (Model 5200, ESA,Inc., Chelmsford, MA) with a dual-electrode microdialysis cell. Each sample (20 μl) was injected into the HPLC consisting of LC-20AT pump, an autosampler, and a C-18 reverse-phase column. The mobile phase consisted of 17% v/v methanol in purified deionized water containing 70 mM KH_2_PO_4_, 0.5 mM EDTA and 8.0 mM sulfonic acid (PH 3.0) and was run at a flow rate of 0.5 ml/min. Peaks were processed by the Azur chromatographic software. Concentrations of dopamine were calculated for each sample.

### Statistics

The differences between multiple group comparisons were made by one-way ANOVA and followed by Fisher’s post-hoc test. Data were presented as mean ± SEM. Significance levels were set to p<0.05 for all comparisons.

## Results

### Derivation of neural progenitors from R1 and TTF-1 cells

**Figure 1:**
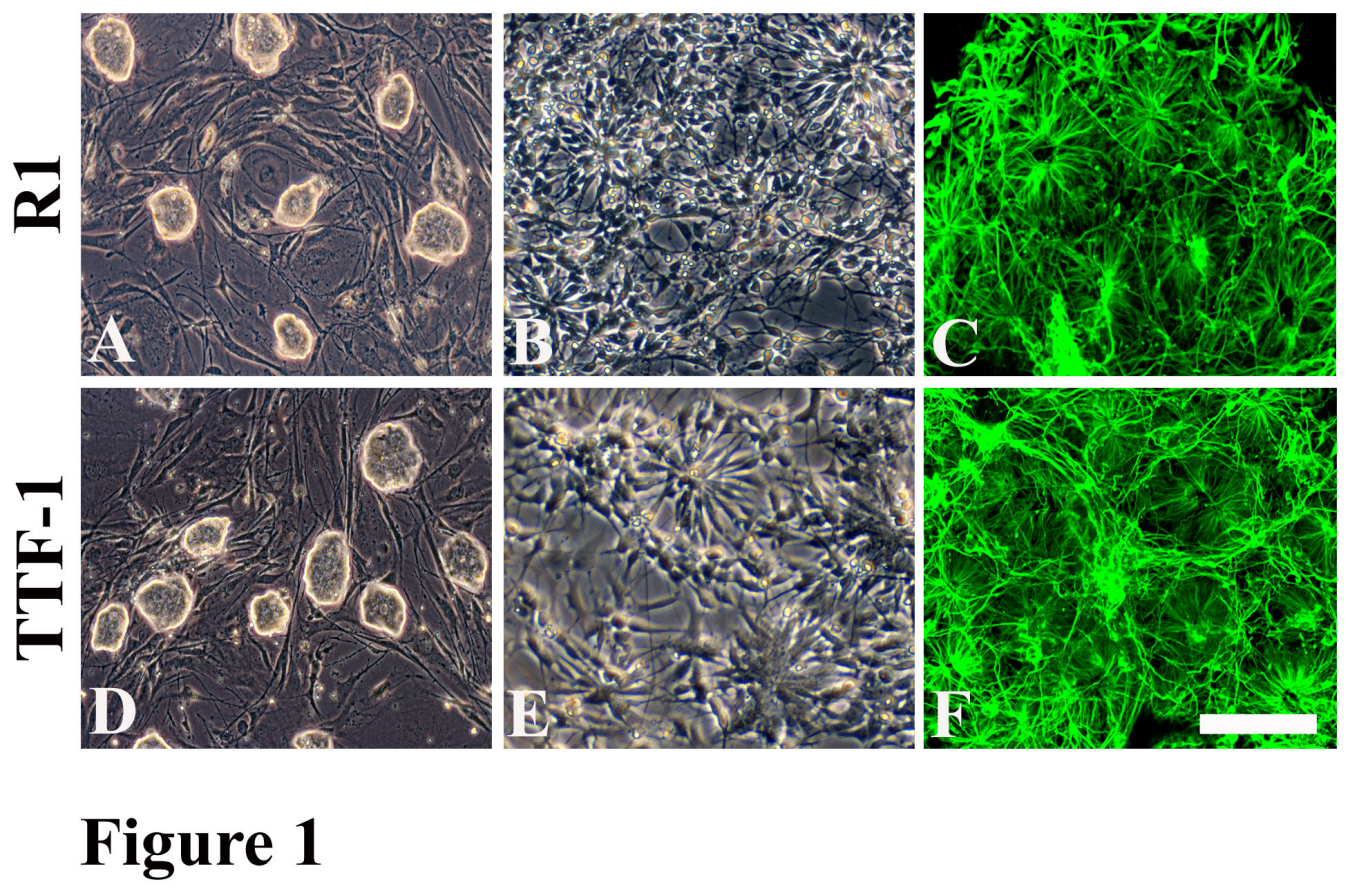
Derivation of neural progenitors from R1 and TTF-1 cells. (A) and (D) Phase contrast image shows that R1 and TTF-1 cells grew as colonies on mitomycin-treated MEF feeder cells. (B) and (E) Phase contrast image shows neural rosettes derived from R1 and TTF-1 cells. (C) and (F) Immunostaining reveals that predominantly cells in rosettes R1 and TTF-1 cells were Nestin positive. Scale bar: 200 µm in A and D; 150 µm in B, C, E, and F.

Both R1 and TTF-1 cells grew as colonies on mitomycin-treated MEF feeder cells (Figures 1A and 1D). After initial expansion on inactivated feeder cells, cell colonies were passaged up to 3 times without feeder cells on gelatin-coated dishes to eliminate contaminating MEFs. Undifferentiated cells were subsequently dissociated into single cells and then grown at 0.5-1×10^5^ cells/ml in aggregate cultures in DFK10 medium to form EBs. After 4 days, EBs were plated on laminin-coated plates and the medium was replaced by DFK10 supplemented with 1% Insulin/Transferrin/Selenium/Fibronectin (ITSFn, GIBCO) medium. Both R1 and TTF-1-derived EBs were shown to efficiently generate neural rosettes (Figures 1B and 1E) in which predominantly cells were positive for nestin staining at 6–10 days post-EB plating (Figures 1C and 1F).

### Dopaminergic differentiation of R1 and TTF-1 cells in SAPNS-3D culture

**Figure 2:**
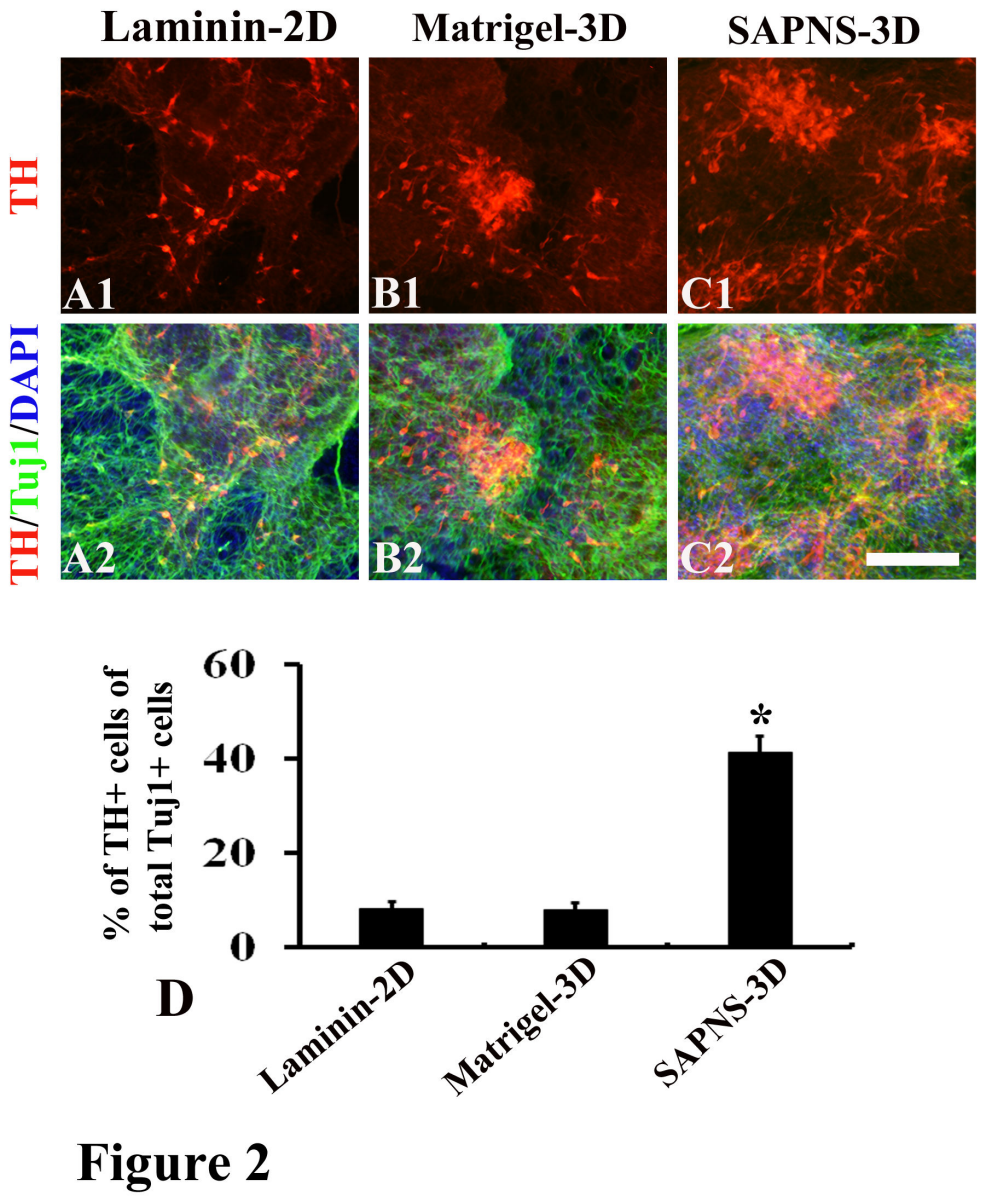
Dopaminergic differentiation of R1 cells was significantly improved in SAPNS-3D culture. (A) Immunocytochemistry for TH and Tuj1 revealed the dopaminergic differentiation of R1 in Laminin-2D culture. (B) Immunocytochemistry for TH and Tuj1 revealed the dopaminergic differentiation of R1 in Matrigel-3D culture. (C) Immunocytochemistry for TH and Tuj1 revealed the dopaminergic differentiation of R1 in SAPNS-3D culture. (D) The percentage of TH-positive cells of total Tuj1-positive cells was 41.5% ± 3.4% in SAPNS-3D culture, which was significantly higher than 8.3% ± 1.4% in the laminin-2D culture and 7.9% ± 1.6% in Matrigel-3D culture. *P<0.001. Scale bar: 150 µm.

**Figure 3:**
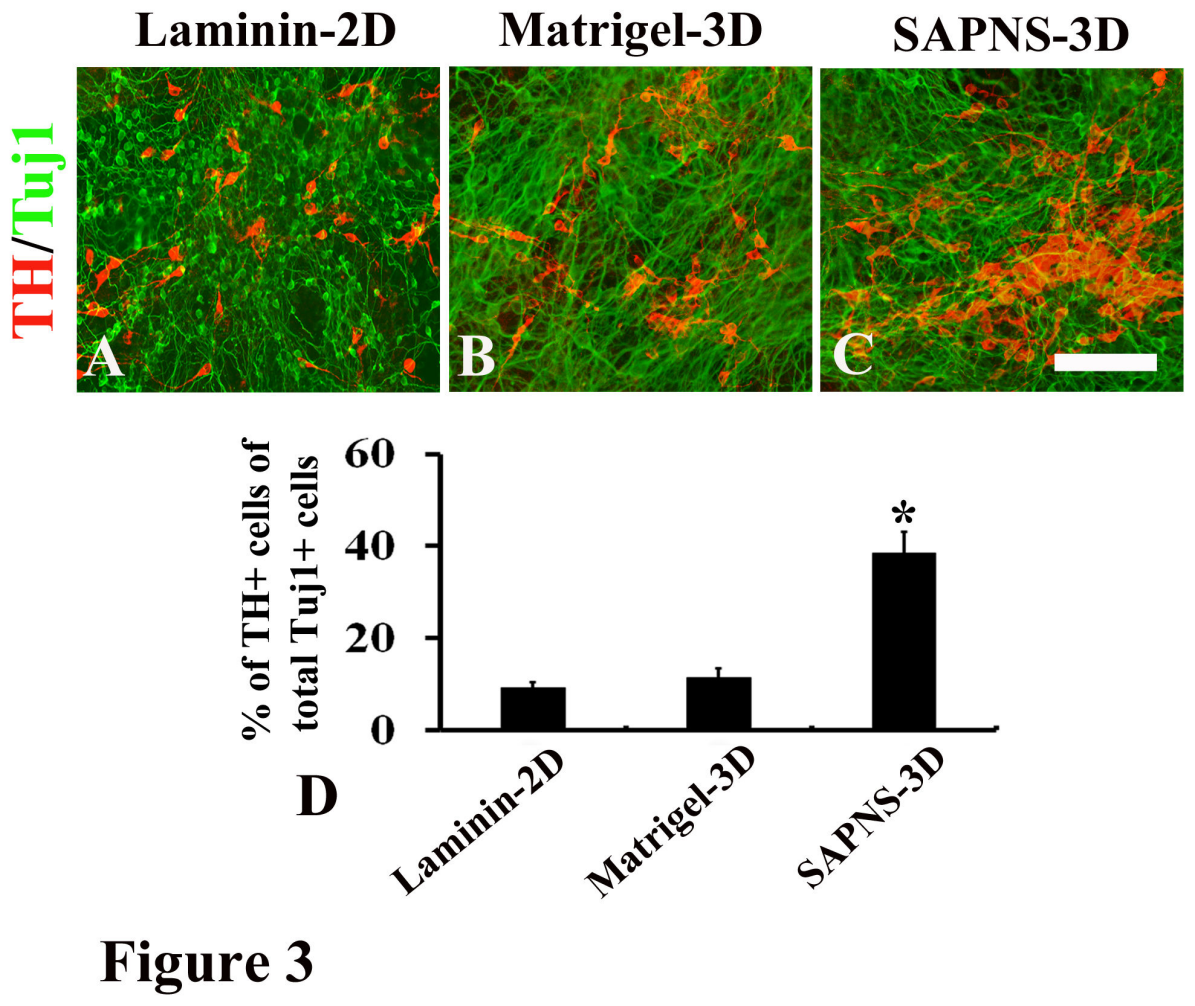
Dopaminergic differentiation of TTF-1 cells was significantly improved in SAPNS-3D culture. (A) Immunocytochemistry for TH and Tuj1 revealed the dopaminergic differentiation of TTF-1 in Laminin-2D culture. (B) Immunocytochemistry for TH and Tuj1 revealed the dopaminergic differentiation of TTF-1 in Matrigel-3D culture. (C) Immunocytochemistry for TH and Tuj1 revealed the dopaminergic differentiation of TTF-1 in SAPNS-3D culture. (D) The percentage of TH-positive cells of total Tuj1-positive cells was 38.5% ± 2.6% in SAPNS-3D culture, which was significantly higher than 9.2% ± 1.2% in the laminin-2D culture and 11.4% ± 2.1% in Matrigel-3D culture. *P<0.001. Scale bar: 150 µm.

We then induced these pluripotent cells-derived neural progenitors towards dopaminergic differentiation in 2D and 3D culture conditions. Neural rosettes were manually selected with a micropipette under a microscope and dissociated by 0.05% trypsin/EDTA. Dissociated cells were encapsulated in SAPNS, Matrigel or directly plated on laminin. After 7 days of exposure to specific midbrain patterning factors (SHH and FGF8b) and followed by 5-10 days of treatment with cAMP and neurotrophic factors, all the cells in the 3 groups did not express pluripotent genes (Figure S1). Successful generation of TH-positive cells was found in all the groups (Figures 2 and 3). TH^+^ cells exhibited typical morphology of dopaminergic neurons with processes and varicose-like structures (Figures 2 and 3). Our study showed that mouse ESCs (R1) generated around 8.3% ± 1.4% TH^+^ cells of total Tuj1^+^ cells in the laminin-coated 2D culture (Figures 2A and 2D), which is consistent with the results in previous reports using 2D culture conditions to induce dopaminergic differentiation of mouse ESCs [1,28,29]. We then observed the cell growth and differentiation in 3D cultures. Cells grew robustly in Matrigel-derived 3D culture (Figure 2B) and an increased cell population was found (data not shown), suggesting that Matrigel is a favorable environment for cell survival and proliferation. The increase in the cell population in Matrigel culture is possibly because of its containing a variety of residual growth factors and undefined constituents. However, dopaminergic differentiation in Matrigel-3D culture did not increase, and the percentage of TH positive cells of total Tuj1-positive cells was 7.9% ± 1.6% (Figures 2B and 2D), which was not different from 8.3% ± 1.4% observed in the conventional laminin-coated 2D culture. This highly indicated that residual growth factors and undefined constituents in Matrigel do not support dopaminergic differentiation. We did not add extra soluble growth factors into RADA16-I peptide hydrogels for SAPNS-derived 3D culture. An interesting finding in the present study was that cells encapsulated in SAPNS grew well (Figure 2C), which indicates that SAPNS supports cell survival in a 3D hydrogel matrix. More interestingly, the dopaminergic differentiation of R1 was pronouncedly enhanced in SAPNS-derived 3D culture condition, and the percentage of TH positive cells of total Tuj1-positive cells was 41.5% ± 3.4%, which was significantly higher than 8.3% ± 1.4% in the conventional laminin-coated 2D culture and 7.9% ± 1.6% in Matrigel-derived 3D culture. We also investigated dopaminergic differentiation of mouse iPSCs in SAPNS-derived 3D culture condition. The cell line TTF-1 was derived from tail tip fibroblasts of adult C57BL/6 mice by transfection of OCT4/SOX2/KLF4 [27]. Similarly to R1 differentiation, dopaminergic differentiation of mouse iPSCs (TTF-1) was greatly improved in SAPNS-derived 3D culture condition compared to that in laminin-coated 2D culture condition or Matrigel-derived 3D culture condition (Figure 3). 

**Figure 4:**
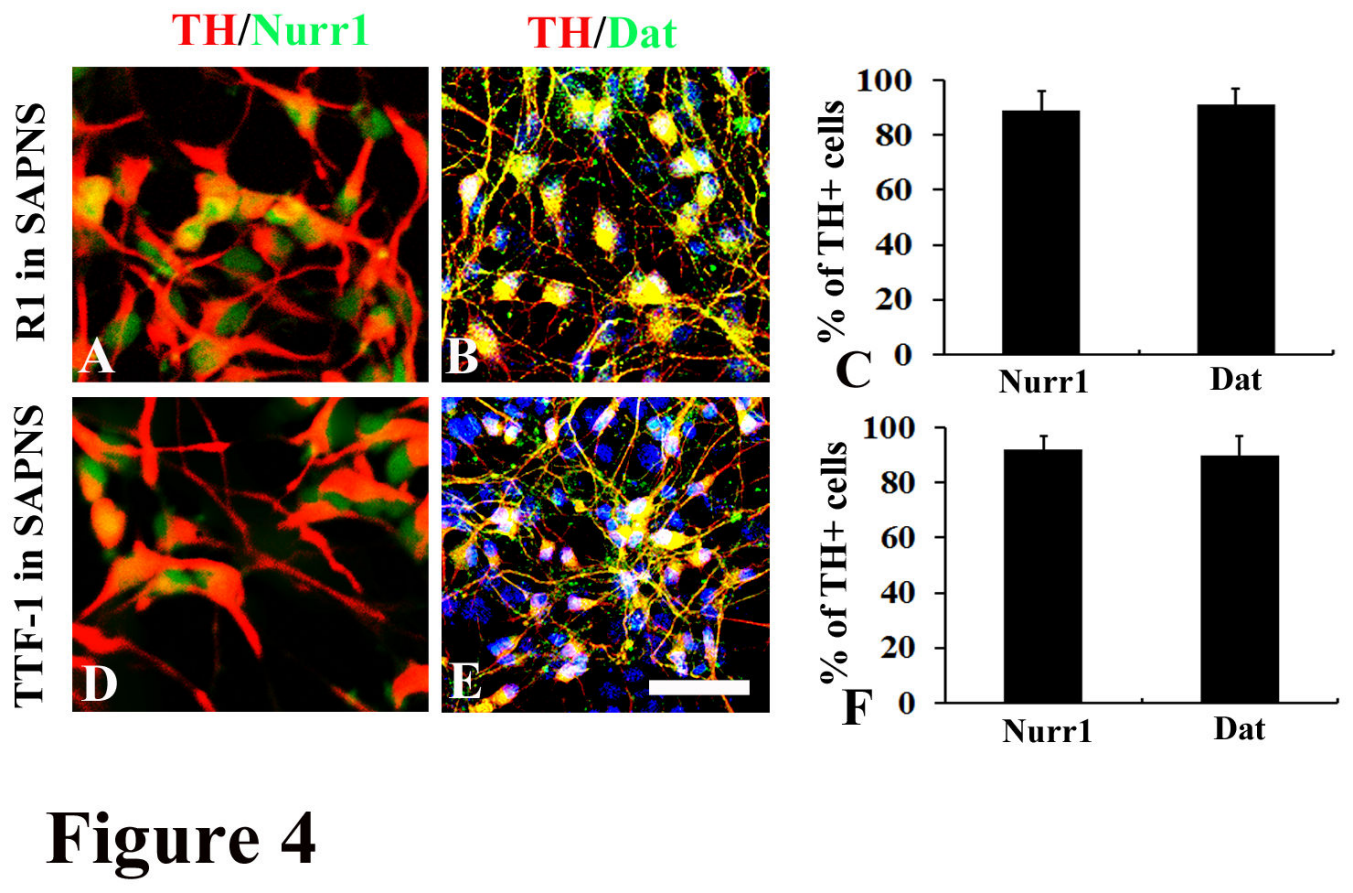
The majority of TH positive neurons co-expressed specific midbrain dopaminergic neuron markers. (A-C) Characterization of R1-derived TH positive neurons in SAPNS. (A) Double immunostaining showed that R1-derived TH positive neurons were co-labeled with Nurrl; (B) Double immunostaining showed that R1-derived TH positive neurons were co-labeled with Dat; (C) The majority of R1-derived TH positive neurons co-expressed Nurr1 or Dat. (D-F) Characterization of TTF-1-derived TH positive neurons in SAPNS. (D) Double immunostaining showed that TTF-1-derived TH positive neurons were co-labeled with Nurrl; (E) Double immunostaining showed that TTF-1-derived TH positive neurons were co-labeled with Dat; (F) The majority of TTF-1-derived TH positive neurons co-expressed Nurr1 or Dat. Scale bar: 100 µm.

We next characterized the subtypes of these TH positive cells derived in SAPNS. Cells were co-immunostained with TH and either Nurrl (dopaminergic neuron marker), Dat (dopaminergic neuron marker), Dbh (adrenergic neuron marker), or Tph2 (serotonergic neuron marker). The majority of R1-derived or TTF-1-derived TH positive cells in SAPNS were co-labeled with dopaminergic neuron markers (R1: 91.5% ± 6.4% TH positive cells were Nurr1 positive and 92.6% ± 7.2% TH positive cells were Dat positive, Figures 4A-C; TTF-1: 93.1% ± 5.3% TH positive cells were Nurr1 positive and 91.7% ± 7.1% TH positive cells were Dat positive, Figures 4D-F). Conversely, few TH positive cells were found to co-express Dbh or Tph2 (data now shown). All these data suggested that the majority of TH positive cells derived in SAPNS were dopaminergic neurons.

### Relative mRNA expression levels of dopaminergic markers in SAPNS-3D culture

**Figure 5:**
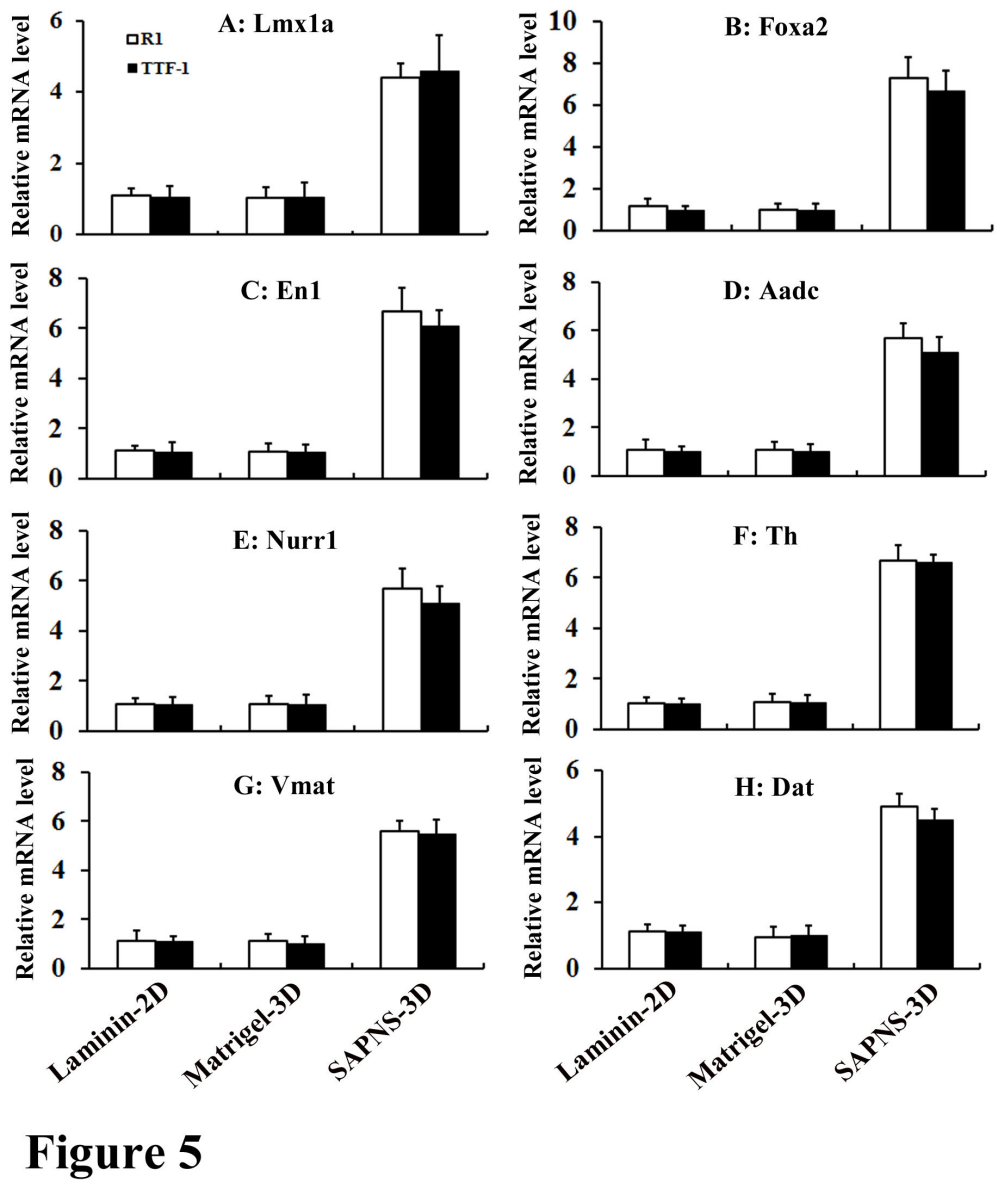
Relative mRNA expression levels of dopaminergic differentiation markers (*Lmx1a*, *Foxa2*, En1, *Aadc*, *Nurr1*, Th, *Vmat2*, and *Dat*) in laminin-2D culture, Matrigel-3D culture and SAPNS-3D culture respectively. Quantitative real-time RT-PCR demonstrated that a 4-fold increase for *Lmx1a* (A), 5-fold increase for *Nurr1* (E), *Vmat2* (G), and *Dat* (H), 6-fold increase for En1 (C) and *Aadc* (D), and 7-fold increase for *Foxa2* (B) and Th (F) were found in both R1 and TTF-1 differentiation when cultured in SAPNS as compared to their expressions in Matrigel or on laminin.

We then carried out investigations on the expression level of a number of dopaminergic markers that play key roles in dopaminergic differentiation and maturation in the midbrain. Lmx1a is one of the earliest intrinsic initiation factors for dopaminergic differentiation and its expression is an entry point into the early molecular pathways of dopaminergic differentiation [30]; Foxa2 and En1 are transcription factors that are highly expressed in dopaminergic precursors [31,32]; Nurr1 is a transcription factor that plays a critical role in the differentiation of midbrain precursors into dopamine neurons [33,34]; Aadc is a multifunctional enzyme that plays an essential role in the biosynthesis of catecholamine neurotransmitters [35,36]; Vmat2, Th, and Dat are typically expressed in midbrain dopaminergic neurons [35,37]. R1 or TTF-1 cells-derived neural progenitors by EB methods were embedded in SAPNS, Matrigel, or directly coated on laminin. After 7 days of exposure to specific midbrain patterning factors (SHH and FGF-8), we collected total RNAs in all the three groups. Quantitative real-time RT-PCR demonstrated that normalized mRNA expression of these genes in the SAPNS group was remarkably higher than that in controls. A 4-12 fold increase for *Lmx1a*, *Foxa2*, *En1, Aadc, Nurr1, Th, Vmat2*, and *Dat* in both R1 and TTF-1 differentiation when cultured in SAPNS was found as compared to their expressions in Matrigel or on laminin (Figure 5). We further investigated the expression of Dbh and Tph2 which code for adrenergic and serotonergic biosynthetic enzymes respectively in SAPNS-derived cells. No expression of Dbh and Tph2 was detected (data not shown), suggesting that there were no adrenergic and serotonergic differentiation in SAPNS culture. 

### Functional analyses of dopaminergic neurons

**Figure 6:**
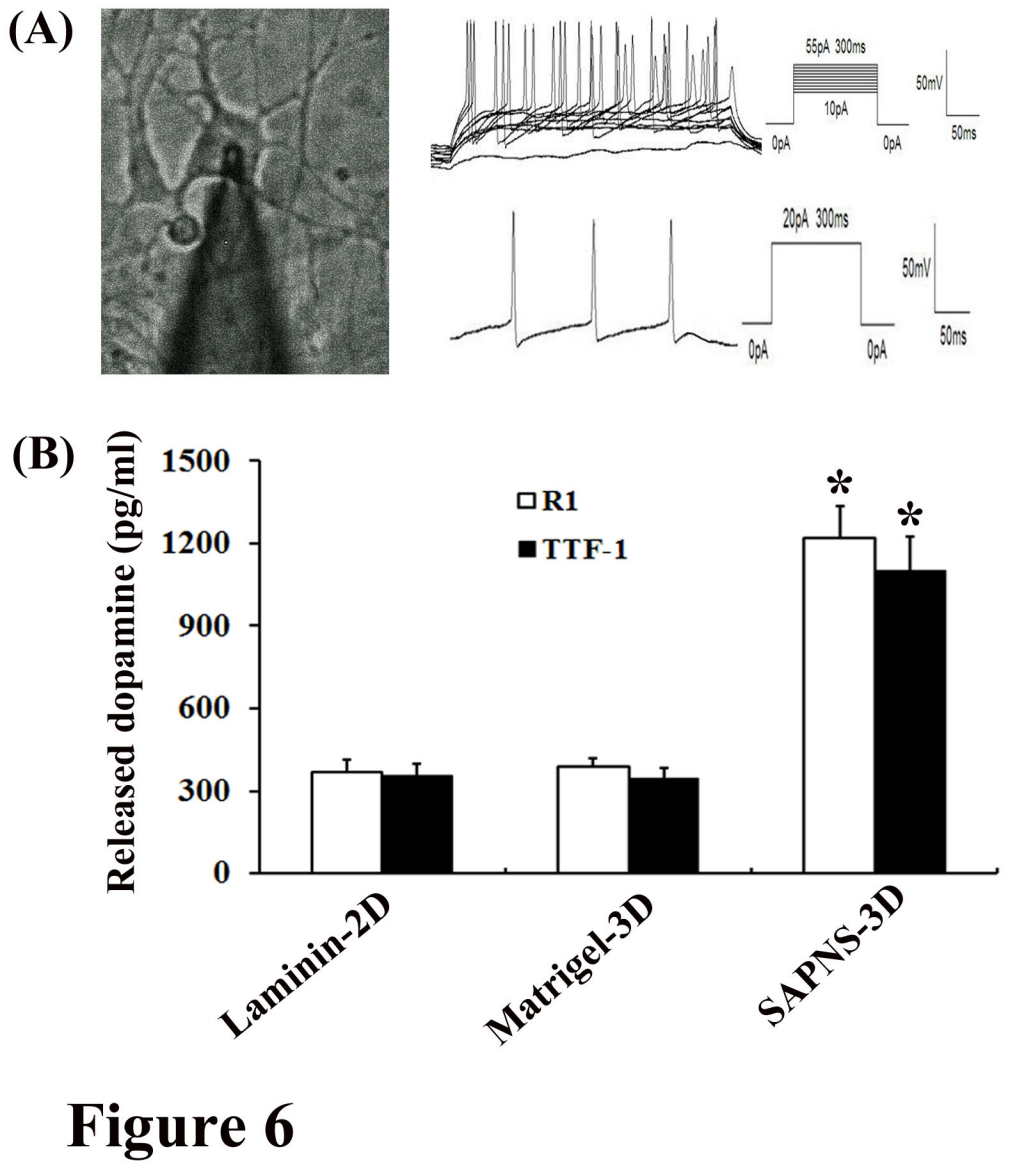
Functional Analyses of dopaminergic neurons. Standard whole-cell patch clamp, current-clamp techniques (A) and dopamine release measurement (B) were performed to explore the biological function of dopaminergic neurons. (A) Current injections (300 ms duration current injections with increasing 5 pA every round) and single current injection (300 ms duration, 20 pA) showing these dopaminergic neurons generated repetitive trains of action potentials. (B) HPLC analyses demonstrate that the dopamine level was increased more than 3-fold in SAPNS culture compared to that in Matrigel culture or laminin culture (* P<0.001 in both R1 and TTF-1 differentiation).

We further investigated functional properties of dopaminergic neurons differentiated in SAPNS. First, standard whole-cell patch clamp, current-clamp techniques were used to study the electrical properties of these dopaminergic neurons. Repetitive traces of action potentials were elicited when a 10 pA current injection was applied and increased 5 pA in every new round until 55 pA was reached, suggesting that they possessed the abilities to produce action potential firing (Figure 6A). Another definitive measure of functional dopaminergic neurons is the production of dopamine. Dopamine release was measured by reverse-phase high-performance liquid chromatography (RP-HPLC) in response to a K+ depolarizing stimulus. Consistent with the increase in the number of TH^+^ neurons in R1 or TTF-1 differentiation cultured in SAPNS, the dopamine level was increased more than 3-fold in SAPNS compared to that in Matrigel culture or laminin culture (p<0.001 in both R1 differentiation and TTF-1 differentiation; Figure 6B). HPLC analyses demonstrated that in R1 differentiation, the evoked release of dopamine was about 1220 ± 115 pg/ml per well in SAPNS-3D culture, while it was 369 ± 47 pg/ml in laminin-2D culture and 387 ± 31 pg/ml in Matrigel-3D culture respectively; in TTF-1 differentiation, the dopamine level was detected at about 1100 ± 126 pg/ml per well in SAPNS-3D culture, and it was 355 ± 41 pg/ml in laminin-2D culture and 346 ± 38 pg/ml in Matrigel-3D culture respectively (Figure 6B).

## Discussion

Dopaminergic differentiation of ESCs gains more and more attentions of worldwide neuroscientists owing to its potential use for neurorestorative therapy for the treatment of Parkinson’s disease. The conventional 2D cell culture on petri dishes with various animal derived substrata such as collagen gels, laminin, and Matrigel is widely used to induce dopaminergic differentiation and it may limit the efficiency in the generation of dopaminergic neurons from ESCs and prevent their application for human therapies. In this study, we reported that a self-assembling peptide made from natural amino acids has a property to generate a true 3D environment for cell growth and differentiation. Mouse ESCs (R1) and mouse iPSCs (TTF-1) embedded in RADA16-I peptide-derived nanofiber scaffolds lead to a marked increase in dopaminergic differentiation compared to the laminin-coated 2D culture or Matrigel-encapsulated 3D culture. These differentiated neurons expressed specific dopaminergic markers and produced appropriate patterns of action potential firing. Consistent with the increase in the number of dopaminergic neurons differentiated from R1 or TTF-1 in the self-assembling peptide nanofiber scaffold (SAPNS), both the expression levels of genes that involve in dopaminergic differentiation and maturation and the dopamine release in SAPNS culture were significantly elevated. The results of the study suggest that SAPNS provides a promising 3D culture system for dopaminergic differentiation.

Diffusion of nutrients and removal of waste products in 2D and 3D cultures are dramatically different and many kinds of cells, including neurons, may alter their metabolism and gene expression patterns in 2D cultures compared to 3D cultures [17]. In addition, cells in 2D culture attach and spread on the surface of culture dishes, possibly leading to the absence of receptors for growth factors, cytokines, and other molecular signals on the attachment side, while cells in a 3D environment where the whole cell body is surrounded by extracellular matrix likely receive more external stimuli than when in contact with coated-2D culture dishes. A 3D environment is possibly more favorable for dopaminergic differentiation than a 2D culture environment. Our study showed that R1 or TTF-1 cells generate more dopaminergic neurons in SAPNS-derived 3D environment possibly due to more surface exposure to specific midbrain patterning factors in a 3D environment than in a 2D environment.

Matrigel is representative of the correct nanoscale biomaterials and widely used in cell cultures. However, it is isolated from mice EHS sarcoma and contains residual growth factors, undefined constituents or non-quantified substances [16,17]. The matrix composition of Matrigel varies from lot to lot, often leading to unstable study results. From time to time, the biochemical pathways observed may be due to unknown cell signaling factors present in the Matrigel. All these indicate that Matrigel is not an ideal biomaterial for a well-controlled 3D culture. In our study, an increased cell population was found in Matrigel-embedded culture possibly because of its containing a variety of adhesion proteins and growth factors. However, dopaminergic differentiation in Matrigel 3D culture did not increase, suggesting that growth factors and cell signaling factors present in the Matrigel do not favor dopaminergic differentiation.

Self-assembling peptides made from natural amino acids can undergo spontaneous self-assembly into nanofibers which are 6–10 nm in diameter and form nanofiber scaffold hydrogels which surround cells in a manner similar to the extracellular matrix [21]. RADA16, also called PuraMatrix, is a self-assembling peptide and extensively used for 3D cell culture, drug delivery, and tissue regeneration [18,24,25,38,39]. Our present study showed that R1 or TTF-1 derivatives embedded in RADA16 peptide-formed nanofiber matrices grew extensively and generated TH^+^ cells with a higher efficiency compared to the 2D culture and 3D culture that employ animal derived materials such as laminin and Matrigel. Meanwhile, a significant elevation in dopamine release was observed in SAPNS culture, suggesting that these TH^+^ cells generated in SAPNS were truly functional. 

Various strategies for improving efficiency of dopaminergic differentiation from ESCs have been employed mainly by optimizing culture conditions, manipulating genetic modification, and modulating intracellular signaling pathways. However, it should be noted that for clinical applications it would be required to eliminate risk of the xenogenic contamination. In addition, although genetic modification could enhance the efficiency of dopaminergic differentiation, this approach may need extra caution to use it for clinical applications due to risks of undesirable side effects. Self-assembling peptides are made of natural amino acids by a chemical peptide synthesis method and each structure is well defined. They possess great potentials in clinical applications due to their excellent biocompatibility and biodegradability. Our present results further provide evidence that self-assembling peptide hydrogels can be used as a promising 3D culture system for dopaminergic differentiation.

## Supporting Information

Figure S1 **The expression profiles of pluripotent genes during differentiation.** (A and B) The pluripotent genes Oct4 (A) and Nanog (B) were highly expressed in R1 and TTF-1 cells at the initial stage. Expression levels decreased dramatically after neural induction. They were below the detection limit after 7 days neural induction. (C and D) The neural progenitor markers Pax6 (C) and Nestin (D) were highly expressed after 7 days neural induction (neural rosette stage). Expression levels of Pax6 and Nestin decreased gradually afterwards and became undetectable after 24 days differentiation.
(TIF)

Table S1 **The sequences of the primers used for evaluating the expression of genes that involve in dopaminergic differentiation or maturation.**
(DOC)
