# Supplementary figures and images for: Self-Assembling Peptide Nanofiber Scaffolds Enhance Dopaminergic Differentiation of Mouse Pluripotent Stem Cells in 3-Dimensional Culture
Source: PLoS One. 2013 Dec 20;8(12):e84504. doi: 10.1371/journal.pone.0084504 (PMC3869843; doi:10.1371/journal.pone.0084504)

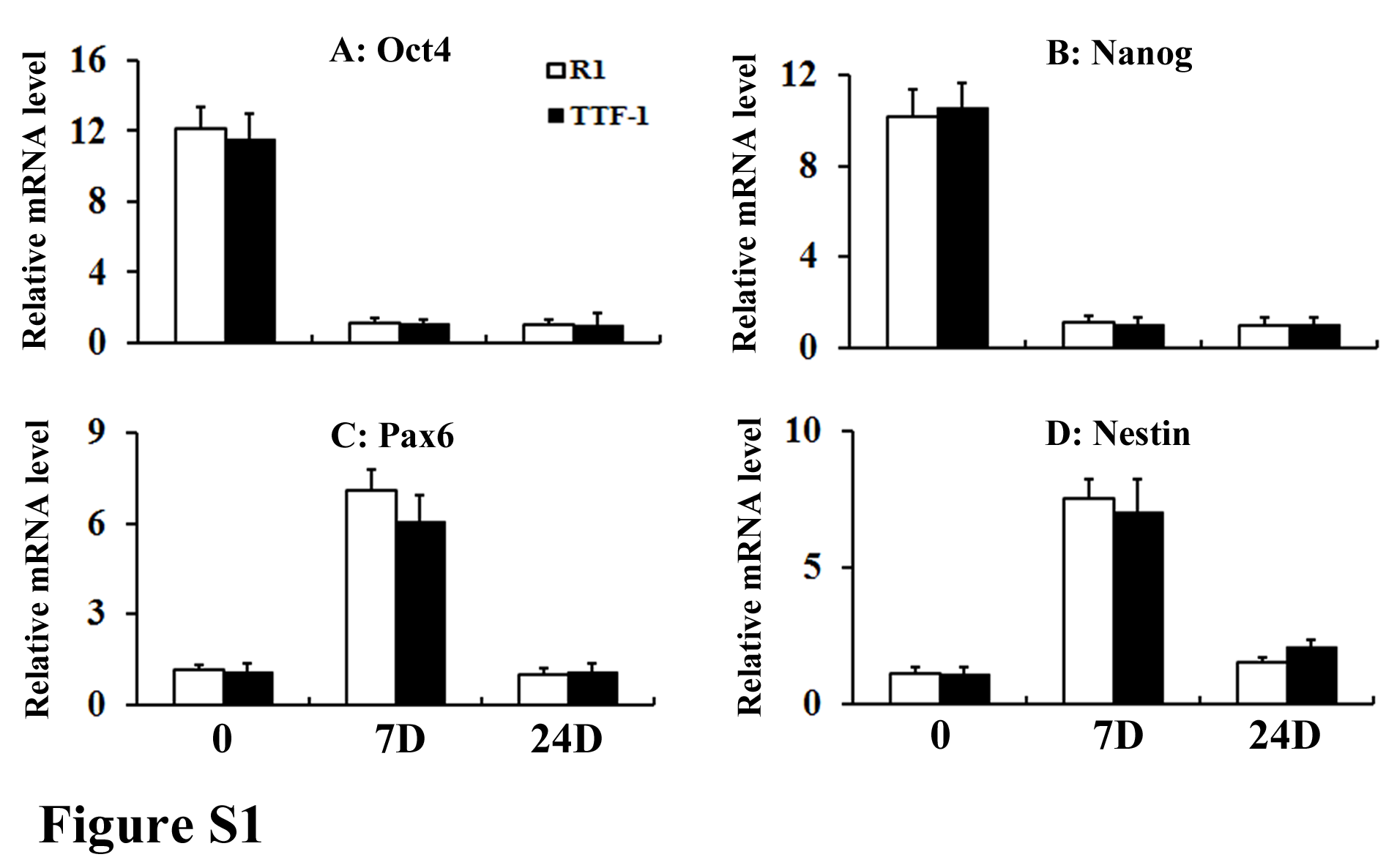

Supplement: Figure S1 — The expression profiles of pluripotent genes during differentiation. (A and B) The pluripotent genes Oct4 (A) and Nanog (B) were highly expressed in R1 and TTF-1 cells at the initial stage. Expression levels decreased dramatically after neural induction. They were below the detection limit after 7 days neural induction. (C and D) The neural progenitor markers Pax6 (C) and Nestin (D) were highly expressed after 7 days neural induction (neural rosette stage). Expression levels of Pax6 and Nestin decreased gradually afterwards and became undetectable after 24 days differentiation. (TIF) [file pone.0084504.s001.tif]
