# Supplementary material for: Self-Assembling Peptide Nanofiber Scaffolds Enhance Dopaminergic Differentiation of Mouse Pluripotent Stem Cells in 3-Dimensional Culture
Source: PLoS One. 2013 Dec 20;8(12):e84504. doi: 10.1371/journal.pone.0084504 (PMC3869843; doi:10.1371/journal.pone.0084504)
Supplement: Table S1 — The sequences of the primers used for evaluating the expression of genes that involve in dopaminergic differentiation or maturation. (DOC) [file pone.0084504.s002.doc]

**Table S1**. Genes and primers

| **Gene** | **Forward primer (5'to 3')** | **Reverse primer (5'to 3')** |
| --- | --- | --- |
| Oct4 | GGATGTGGTTCGAGTATGGTTC | GTAGAGTGTGGTGAAGTGGG |
| Nanog | CCACCAGGTGAAATATGAGAC | TATTTGGAAGAAGGAAGGAACC |
| Th | AAGGACAAGCTCAGGAACTATG | GCATTTAGCTAATGGCACTCAG |
| Vmat2 | GTATGCTATCGGTCCCTCTG | GAGTGTACATCTTTGTCTTAATGG |
| Dat | GGTCCTTCCGAGAGAAACTG | TCTCCTTCCACTTTACACCAAC |
| Pax6 | ACACCTGTCTCCTCCTTCAC | GGTTGCATAGGCAGGTTGTTTG |
| Nestin | AAGTGGCTACATACAGGACTC | TGAGGACAGGGAGCACAGA |
| Aadc | AGGGCAGAGAAAGAATGAAAGCA | GGAGTGGTAGTTATTTTTCTCTTTCCAGTTT |
| Tph2 | CGCTTACTTTGTTTCGGACAG | TCTGCGTGTAGGGGTTGAAG |
| Dbh | TTCCAATGTGCAGCTGAGTC | TATCTTCCGTGGGTTGTGGT |
| Lmx1a | AGCGAGCCAAGATGAAGAAG | GAGTTGTAGACGCTCTGTTC |
| En1 | GTCCGTCCTCTGGTCCAC | CGTGATATAGCGGTTTGCCTG |
| Foxa2 | GTACTCCAGGCCTATTATGAAC | TTCCTCAAAGCTCTCCCAAAG |
| Nurr1 | GACCCGGGCTCCTCTGCTCC | GGCATGGCTTCAGCAGAGTTATTGG |
| *β*-Actin | TCATGAAGTGTGACGTGGACATC | TGTTGCATTTGCGGGGACGATG |
